# Supplementary material for: Healthy living environments create healthy physical activity-related habits (ELEVATE): A protocol for a smartphone-based mixed-method study
Source: PLoS One. 2026 May 11;21(5):e0348889. doi: 10.1371/journal.pone.0348889 (PMC13160327; doi:10.1371/journal.pone.0348889)
Supplement: Attachment — Submitted filename: Response_to_Reviewers.docx [file pone.0348889.s001.docx]

**Response (PONE-D-26-07970)**

We thank the editor, Prof. Dong Liu, and the two reviewers for their insightful and constructive feedback, which has improved our manuscript. Our point-by-point responses to their comments are provided below.

**Journal Requirements**

When submitting your revision, we need you to address these additional requirements.

1.Please ensure that your manuscript meets PLOS ONE's style requirements, including those for file naming. The PLOS ONE style templates can be found at

<https://journals.plos.org/plosone/s/file?id=wjVg/PLOSOne_formatting_sample_main_body.pdf> and

Response: Done. The revised manuscript follows the style requirements.

[https://journals.plos.org/plosone/s/file?id=ba62/PLOSOne_formatting_sample_title_authors_affiliations.pdf](https://eur03.safelinks.protection.outlook.com/?url=https%3A%2F%2Ftrack.editorialmanager.com%2FCL0%2Fhttps%3A%252F%252Fjournals.plos.org%252Fplosone%252Fs%252Ffile%253Fid%3Dba62%252FPLOSOne_formatting_sample_title_authors_affiliations.pdf%2F1%2F010f019d2d2edd12-15973f6e-7087-4ad8-abe7-b1ff7ac18fba-000000%2FvxR_GMlYLxcuc4f-7br_9xpDCAwTG9L_kxIQifvsMA0%3D256&data=05%7C02%7Cm.helbich%40uu.nl%7C35b898c9d44c483161ae08de8baaef27%7Cd72758a0a4464e0fa0aa4bf95a4a10e7%7C0%7C0%7C639101763939403714%7CUnknown%7CTWFpbGZsb3d8eyJFbXB0eU1hcGkiOnRydWUsIlYiOiIwLjAuMDAwMCIsIlAiOiJXaW4zMiIsIkFOIjoiTWFpbCIsIldUIjoyfQ%3D%3D%7C0%7C%7C%7C&sdata=A2bZHbDSDt%2BXVEgPzGSiGkiQoS%2BHyOp2gRk%2FLbDBYpc%3D&reserved=0)

Response: Done. The revised manuscript follows the style requirements.

2. In the ethics statement in the Methods, you have specified that verbal consent was obtained. Please provide additional details regarding how this consent was documented and witnessed, and state whether this was approved by the IRB.

Response: Done. The subjects agree verbally, but they still need to sign the consent form. The sentence was rephrased as follows: “Participants will provide consent via the smartphone application by selecting a checkbox; the application will automatically record the timestamp of consent. Separate consent will be obtained for participation in the focus group, for audio recording of the walk-along interview, and for the use of the transcript solely for analytical purposes. A separate consent form must be completed and signed for each component. […] The ethical and data protection aspects of ELEVATE were reviewed and approved by the Ethics Review Board of Utrecht University (Geo-25-0165).”

3. Please note that funding information should not appear in any section or other areas of your manuscript. We will only publish funding information present in the Funding Statement section of the online submission form. Please remove any funding-related text from the manuscript.

Response: Confirmed. We only mention the funding in the funding statement.

4. Thank you for stating the following in the Competing Interests section:

“The authors have declared that no competing interests exist.”

We note that one or more of the authors are employed by a commercial company: Studio Bereikbaar, De Boom en het Meer, Track Landscapes and Bureau Buiten.

1.        Please provide an amended Funding Statement declaring this commercial affiliation, as well as a statement regarding the Role of Funders in your study. If the funding organization did not play a role in the study design, data collection and analysis, decision to publish, or preparation of the manuscript and only provided financial support in the form of authors' salaries and/or research materials, please review your statements relating to the author contributions, and ensure you have specifically and accurately indicated the role(s) that these authors had in your study. You can update author roles in the Author Contributions section of the online submission form.

Please also include the following statement within your amended Funding Statement.

“The funder provided support in the form of salaries for authors [insert relevant initials], but did not have any additional role in the study design, data collection and analysis, decision to publish, or preparation of the manuscript. The specific roles of these authors are articulated in the ‘author contributions’ section.”

If your commercial affiliation did play a role in your study, please state and explain this role within your updated Funding Statement.

Response: Done. Adjusted as follows: “The ELEVATE project is funded by Health-Holland, Top Sector Life Sciences & Health (grant number HH-LoM-033). The project underwent independent peer review as part of the funding process. All project partners, including academic and commercial organizations (Studio Bereikbaar, De Boom en het Meer, Track Landscapes, and Bureau Buiten), receive funding through this consortium. Health-Holland provides financial support in the form of authors’ salaries and/or research materials but does not have a direct role in the study design, data collection and analysis, decision to publish, or preparation of the manuscript. The commercial partners were involved in developing the research proposal and will contribute to the conduct of the study. The specific roles of all authors, including those affiliated with commercial organizations, are described in the Author Contributions section.” The author's contributions statement accurately reflects each person's contributions.

2. Please also provide an updated Competing Interests Statement declaring this commercial affiliation along with any other relevant declarations relating to employment, consultancy, patents, products in development, or marketed products, etc.

Within your Competing Interests Statement, please confirm that this commercial affiliation does not alter your adherence to all PLOS ONE policies on sharing data and materials by including the following statement: "This does not alter our adherence to  PLOS ONE policies on sharing data and materials.” (as detailed online in our guide for authors <http://journals.plos.org/plosone/s/competing-interests>). If this adherence statement is not accurate and  there are restrictions on sharing of data and/or materials, please state these. Please note that we cannot proceed with consideration of your article until this information has been declared.

Please include both an updated Funding Statement and Competing Interests Statement in your cover letter. We will change the online submission form on your behalf.

Response: Done. We updated the statement as follows: “The authors declare that some authors are employed by commercial organizations (Studio Bereikbaar, De Boom en het Meer, Track Landscapes, and Bureau Buiten). There are no patents, products in development, or marketed products associated with this research. This does not alter our adherence to PLOS ONE policies on data and materials sharing.”

5. Your ethics statement should only appear in the Methods section of your manuscript. If your ethics statement is written in any section besides the Methods, please delete it from any other section.

Response: Corrected. We copied the ethics and data management section to the Methods section as requested.

6. We note that Figure 2 in your submission contain copyrighted images. All PLOS content is published under the Creative Commons Attribution License (CC BY 4.0), which means that the manuscript, images, and Supporting Information files will be freely available online, and any third party is permitted to access, download, copy, distribute, and use these materials in any way, even commercially, with proper attribution. For more information, see our copyright guidelines: <http://journals.plos.org/plosone/s/licenses-and-copyright>.  We require you to either (1) present written permission from the copyright holder to publish these figures specifically under the CC BY 4.0 license, or (2) remove the figures from your submission:

 1. You may seek permission from the original copyright holder of Figure 2 to publish the content specifically under the CC BY 4.0 license. We recommend that you contact the original copyright holder with the Content Permission Form (<http://journals.plos.org/plosone/s/file?id=7c09/content-permission-form.pdf>) and the following text:

“I request permission for the open-access journal PLOS ONE to publish XXX under the Creative Commons Attribution License (CCAL) CC BY 4.0 (<http://creativecommons.org/licenses/by/4.0/>). Please be aware that this license allows unrestricted use and distribution, even commercially, by third parties. Please reply and provide explicit written permission to publish XXX under a CC BY license and complete the attached form.”

 Please upload the completed Content Permission Form or other proof of granted permissions as an "Other" file with your submission.  In the figure caption of the copyrighted figure, please include the following text: “Reprinted from [ref] under a CC BY license, with permission from [name of publisher], original copyright [original copyright year].”

2. If you are unable to obtain permission from the original copyright holder to publish these figures under the CC BY 4.0 license or if the copyright holder’s requirements are incompatible with the CC BY 4.0 license, please either i) remove the figure or ii) supply a replacement figure that complies with the CC BY 4.0 license. Please check copyright information on all replacement figures and update the figure caption with source information. If applicable, please specify in the figure caption text when a figure is similar but not identical to the original image and is therefore for illustrative purposes only.

Response: Added. The figure images are not sourced from copyrighted material. They illustrate a conceptual AI-driven recommendation system; no such system has been developed to date. The images were generated using ChatGPT-4.5 based on author-defined prompts and subsequent editing to reflect plausible real-world conditions. The authors take full responsibility for these generated images, which are shared under the CC BY 4.0 license. We added the following to the figure caption: “The image is for illustrative purposes and was generated using ChatGPT-4.5 based on author-defined prompts and subsequent editing to reflect plausible real-world conditions.”

7. If the reviewer comments include a recommendation to cite specific previously published works, please review and evaluate these publications to determine whether they are relevant and should be cited. There is no requirement to cite these works unless the editor has indicated otherwise.

Response: Done. Thanks for pointing this out.

**Reviewers' comments**

1. Does the manuscript provide a valid rationale for the proposed study, with clearly identified and justified research questions? The research question outlined is expected to address a valid academic problem or topic and contribute to the base of knowledge in the field.

Reviewer #1: Yes	Reviewer #2: Yes

2. Is the protocol technically sound and planned in a manner that will lead to a meaningful outcome and allow testing the stated hypotheses? The manuscript should describe the methods in sufficient detail to prevent undisclosed flexibility in the experimental procedure or analysis pipeline, including sufficient outcome-neutral conditions (e.g. necessary controls, absence of floor or ceiling effects) to test the proposed hypotheses and a statistical power analysis where applicable. As there may be aspects of the methodology and analysis which can only be refined once the work is undertaken, authors should outline potential assumptions and explicitly describe what aspects of the proposed analyses, if any, are exploratory.

Reviewer #1: Yes	Reviewer #2: Yes

3. Is the methodology feasible and described in sufficient detail to allow the work to be replicable? Descriptions of methods and materials in the protocol should be reported in sufficient detail for another researcher to reproduce all experiments and analyses. The protocol should describe the appropriate controls, sample size calculations, and replication needed to ensure that the data are robust and reproducible.

Reviewer #1: Yes	Reviewer #2: Yes

4. Have the authors described where all data underlying the findings will be made available when the study is complete? The [PLOS Data policy](https://eur03.safelinks.protection.outlook.com/?url=https%3A%2F%2Ftrack.editorialmanager.com%2FCL0%2Fhttps%3A%252F%252Fjournals.plos.org%252Fplosone%252Fs%252Fmaterials-and-software-sharing%2F1%2F010f019d2d2edd12-15973f6e-7087-4ad8-abe7-b1ff7ac18fba-000000%2FRXStKBpnpCxUFWBIlZMRfaJtTdGqq_WLdiIij1iM-DU%3D256&data=05%7C02%7Cm.helbich%40uu.nl%7C35b898c9d44c483161ae08de8baaef27%7Cd72758a0a4464e0fa0aa4bf95a4a10e7%7C0%7C0%7C639101763939430224%7CUnknown%7CTWFpbGZsb3d8eyJFbXB0eU1hcGkiOnRydWUsIlYiOiIwLjAuMDAwMCIsIlAiOiJXaW4zMiIsIkFOIjoiTWFpbCIsIldUIjoyfQ%3D%3D%7C0%7C%7C%7C&sdata=0c9s49D11JxNTt6tJjMynRmBBYpvAeYWJ3WaGid7Stg%3D&reserved=0) requires authors to make all data underlying the findings described in their manuscript fully available without restriction, with rare exception, at the time of publication. The data should be provided as part of the manuscript or its supporting information, or deposited to a public repository. For example, in addition to summary statistics, the data points behind means, medians and variance measures should be available. If there are restrictions on publicly sharing data—e.g. participant privacy or use of data from a third party—those must be specified.

Reviewer #1: Yes	Reviewer #2: Yes

5. Is the manuscript presented in an intelligible fashion and written in standard English? PLOS ONE does not copyedit accepted manuscripts, so the language in submitted articles must be clear, correct, and unambiguous. Any typographical or grammatical errors should be corrected at revision, so please note any specific errors here.

Reviewer #1: Yes	Reviewer #2: Yes

6. Review Comments to the Author. Please use the space provided to explain your answers to the questions above and, if applicable, provide comments about issues authors must address before this protocol can be accepted for publication. You may also include additional comments for the author, including concerns about research or publication ethics.

You may also provide optional suggestions and comments to authors that they might find helpful in planning their study. (Please upload your review as an attachment if it exceeds 20,000 characters)

Response: Please see our response below.

**Reviewer #1**

Thank you for inviting me to review this study protocol that seeks to an important public health and urban planning question: how micro-scale streetscape characteristics shape walking-related physical activity and how these relationships may differ across socioeconomic groups. The protocol is ambitious and has several notable strengths, including the integration of smartphone-based GPS and step-count data, detailed environmental exposure assessment using street-view imagery and GIS, and a mixed-methods design that combines quantitative analysis with qualitative interviews and co-creation elements. The emphasis on equity is also a clear strength. Overall, this is a strong and interesting protocol with a solid rationale, a well-designed mixed-methods approach, and clear relevance for both research and practice. Below please see my specific concerns.

Response: We sincerely thank you for your thorough review of this manuscript and for your constructive comments.

1. In the data analysis plan section, the authors explain that GPS trajectories will be cleaned and matched with environmental indicators, which is helpful. It would further strengthen the protocol if the authors briefly clarify how exposures will be estimated. A short clarification here would improve transparency and reproducibility.

Response: Done. We have now clarified the exposure assessment approach in the Data Analysis Plan section, specifying how GPS trajectories will be linked to micro-scale environmental indicators across space and time. The following was added: “Accelerometer and GPS data will be matched and integrated with micro-scale built and natural environmental indicators using GIS. For recorded locations, exposures will be assigned based on street segment characteristics, with optional buffering (e.g., 25 m) to account for positional uncertainty. Environmental indicators will then be aggregated to the temporal resolution of the outcome (e.g., half-hour intervals) by computing time-weighted averages along individual mobility paths.”

2. The manuscript already addresses ethics, pseudonymization, GDPR compliance, and secure storage, which is very good. Because GPS trajectories are particularly sensitive, it would be helpful to add one or two more sentences on practical geoprivacy protections, for example how home/work locations or highly identifying path information will be handled in derived datasets and outputs.

Response: Done. We added: “Raw GPS data will not be published, as sensitive locations (e.g., home and work) could be inferred. To protect geoprivacy, data will be geomasked (e.g., by adding random noise) and/or aggregated to coarser spatial units prior to dissemination, preventing re-identification. ”

3. For the qualitative component, a brief note on coder procedures and how qualitative and quantitative findings will be integrated would make the protocol even clearer.

Response: Done. We added the following: “The results will, amongst others, identify participant-defined environmental factors influencing walking practices and experiences. Qualitative findings on the lived experiences of streetscapes will also be mapped against quantitative project findings to contextualize and further explain the latter. A purposive sample, comprising approximately 25% of individuals from marginalized groups (e.g., those with low socioeconomic status or limited mobility), will be recruited through neighborhood ambassadors or trusted community intermediaries [52]. Based on a review [53], data saturation is expected to occur with approximately 20 participants.”

“The on-site and walk-along interviews will be fully transcribed and analyzed using a thematic analysis approach. With the assistance of NVivo software, multiple rounds of deductive and inductive coding will be applied to identify overarching themes [78] – including recurring perceptions, experiences, and interactions with the features of the built and natural environment. A codebook will be developed to guarantee transparency, consistency and validity throughout the data analysis process. Resulting themes will be compared across participants and urban settings (e.g., neighborhoods, streetscapes) to highlight both shared patterns and place as well as participant-specific variations. The qualitative analysis will be set up in alignment with a reporting standard [79]. At the beginning of the project, on-site interviews will be organized to explore environmental factors influencing walking practices and experiences that need to be addressed with the baseline survey. Later in the project, walk-along interviews will be organized, and the results will be used to contextualize and ‘spatially ground’ statistical results as well as to further explain and validate quantitative findings.”

**Reviewer #2:**

This research proposal employs a smartphone-based mixed-methods approach to investigate the association between urban streetscape environments and walking-related physical activity (WPA). The topic carries practical significance for both public health and urban planning, and puts forward systematic solutions to critical gaps in existing research regarding data collection, environmental assessment scales, study design, and research translation. The following revision suggestions are provided for the authors’reference.

Response: Thank you for taking the time to review this manuscript. We appreciate your comments.

1. (Introduction) (Background and rationale): On page 3, it is mentioned that “threshold effects may exist in these associations”, yet no specific examples of environmental characteristic thresholds (e.g., thresholds for green space coverage or street connectivity) are cited from existing literature. It is recommended to supplement 1–2 typical cases to lay a more solid foundation for subsequent research hypotheses and analyses.

Response: Done. We added the following: “For example, an international study found that walking is positively associated with intersection density up to about 100 intersections per km2, after which gains level off [13].”

2. Objectives and hypotheses: Hypothesis 4 states that “co-created urban streetscape design principles will increase WPA usage in public spaces”, but does not specify the methods for testing this hypothesis (e.g., pre–post comparison, simulation application evaluation). It is recommended to add such details to align research hypotheses with subsequent methods.

Response: After reviewing your comments and revisiting the section, we decided to remove the example hypotheses, as they largely overlapped with the study aims. Please note that this study does not include pre-post comparisons.

3. (Study site): Only the research advantages of Amsterdam are described (clear socioeconomic and health inequalities, abundant streetscape data), without addressing the potential impacts of the city’s unique streetscape characteristics (e.g., high-density neighbourhoods, extensive bicycle lane networks) on WPA. It is recommended to supplement this background and clarify the generalisability boundaries of the study findings.

Response: We agree with the reviewer that the results may not be generalizable across all urban contexts. To acknowledge this limitation, we have added a statement in the Discussion section emphasizing this point. Please see our response to Comment #7.

4. Recruitment and survey design: It is mentioned that “community ambassadors will be engaged to recruit vulnerable groups”, but the selection criteria for community ambassadors and recruitment incentives are not specified. It is recommended to supplement these details to ensure recruitment feasibility and sample diversity.

Response: Done. We added the requested information: “Ambassadors will be drawn from existing consortium partner networks and engaged purposively based on their local knowledge and community connections.” No additional recruitment incentives beyond those for study participation will be used.

5. Data analysis plan: It is noted that “directed acyclic graphs (DAGs) will be used to identify potential covariates from questionnaires”, yet the core variable set and construction method for DAG development are not explained; this content should be added. Meanwhile, the analytical division of labour between mixed-effects negative binomial regression models and XGBoost models is not clarified (e.g., the former for testing linear associations and the latter for exploring nonlinear and interactive effects). It is recommended to refine the logic of model application.

Response: Done. The following was added: “Directed acyclic graphs, informed by theory and prior evidence, will be used to identify minimally sufficient sets of covariates (e.g., age, income) for adjustment in the regression models [71,72].”

We have clarified the analytical approach, specifying that mixed-effects models are used for primary inference, while machine learning models are applied exploratorily. The text has been revised as follows: “We will fit covariate-adjusted mixed negative binomial regression models as the primary inferential approach to account for repeated measurements of WPA within individuals [70]. […] Machine learning models (e.g., XGBoost) will be applied exploratorily to capture nonlinearity, uncover variable importance, and model complex variable interactions. Shapley values will ease model interpretation (e.g., uncover variable importance and non-linear associations) [74].”

6. Practical and operational considerations: It is mentioned that “smartphone data collection is affected by battery consumption and signal loss”, but no specific technical solutions are proposed (e.g., low-power collection modes, offline data storage), with only pilot testing mentioned. It is recommended to supplement targeted mitigation measures to enhance the study’s operability.

Response: We appreciate this comment. However, without the results of the pilot test, outlining specific mitigation strategies for all potential scenarios would be premature. We note that TU/e has extensive experience in developing sensing solutions in previous projects, which is expected to address most issues related to the app’s operability.

7. (Conclusions): The conclusion only emphasises the research value for “equitable, walkable urban environments” without explicitly acknowledging study limitations (e.g., the sample being limited to Amsterdam, impacts of urban environmental changes during the study period). It is recommended to add a limitation analysis to make the conclusions more objective.

Response: Done. We have added a limitation section: “While ELEVATE is among the first European mixed-method efforts to assess how objectively measured and subjectively experienced micro-environments shape daily WPA, several limitations should be acknowledged. First, smartphone-derived step counts may be less accurate than those from dedicated accelerometers, as they depend on participants’ adherence to wearing protocols. Despite clear instructions, inconsistent device carriage and variability across devices and operating systems may result in misestimation of WPA. Besides careful validation against research-grade accelerometers, we may apply wear-time thresholds and restrict analyses to participants with high adherence to reduce this issue. Second, smartphone-based data collection may lead to selective participation and lower response rates, particularly among individuals with lower socioeconomic status and limited digital literacy, potentially affecting sample representativeness. To mitigate this risk, we will continuously cross-compare the sample characteristics with population data and, if needed, target specific groups. Third, as is typical for travel surveys, a one-week observation period may not capture seasonal or longer-term variability in WPA and environmental exposures. Fourth, Amsterdam’s high urban density, mixed land use, and extensive active transport infrastructure may shape activity patterns, making the findings most applicable to similar urban contexts and warranting caution in lower-density or car-dependent settings. Although generalizability may be limited due to data collection in Amsterdam only, we will share tools and methods to facilitate replication in other settings. Fifths, short-term changes in the urban environment during the study period (e.g., construction, road closures, or new infrastructure) may influence WPA independently of the measured exposures. As these temporal dynamics are not captured in our geodata, this may introduce minor exposure misclassification. Sixth, the observational design precludes causal inference regarding associations between micro-scale environments and WPA.”

PLOS authors have the option to publish the peer review history of their article ([what does this mean?](https://eur03.safelinks.protection.outlook.com/?url=https%3A%2F%2Ftrack.editorialmanager.com%2FCL0%2Fhttps%3A%252F%252Fjournals.plos.org%252Fplosone%252Fs%252Feditorial-and-peer-review-process%2523loc-peer-review-history%2F1%2F010f019d2d2edd12-15973f6e-7087-4ad8-abe7-b1ff7ac18fba-000000%2FWLn486nXxNVrJxz3Jq3Bo_nmvpBqWYqCTi8s2SywWuM%3D256&data=05%7C02%7Cm.helbich%40uu.nl%7C35b898c9d44c483161ae08de8baaef27%7Cd72758a0a4464e0fa0aa4bf95a4a10e7%7C0%7C0%7C639101763939458020%7CUnknown%7CTWFpbGZsb3d8eyJFbXB0eU1hcGkiOnRydWUsIlYiOiIwLjAuMDAwMCIsIlAiOiJXaW4zMiIsIkFOIjoiTWFpbCIsIldUIjoyfQ%3D%3D%7C0%7C%7C%7C&sdata=HAOEiXg2nW%2FBa%2BjuSZ9r2Yf5Kwxv71dpu8%2F9H9aAEBc%3D&reserved=0)). If published, this will include your full peer review and any attached files. If you choose “no”, your identity will remain anonymous but your review may still be made public.

**Do you want your identity to be public for this peer review?** For information about this choice, including consent withdrawal, please see our [Privacy Policy](https://eur03.safelinks.protection.outlook.com/?url=https%3A%2F%2Ftrack.editorialmanager.com%2FCL0%2Fhttps%3A%252F%252Fwww.plos.org%252Fprivacy-policy%2F1%2F010f019d2d2edd12-15973f6e-7087-4ad8-abe7-b1ff7ac18fba-000000%2FBWtKgrC7Xgl4fV1GccULjvcR-BvjZWVHi3d5y6GUOEY%3D256&data=05%7C02%7Cm.helbich%40uu.nl%7C35b898c9d44c483161ae08de8baaef27%7Cd72758a0a4464e0fa0aa4bf95a4a10e7%7C0%7C0%7C639101763939483022%7CUnknown%7CTWFpbGZsb3d8eyJFbXB0eU1hcGkiOnRydWUsIlYiOiIwLjAuMDAwMCIsIlAiOiJXaW4zMiIsIkFOIjoiTWFpbCIsIldUIjoyfQ%3D%3D%7C0%7C%7C%7C&sdata=JpXLpZEGQCbxQIXLGGD44eKhQbheQh7PmqtbkhdQwK8%3D&reserved=0).

Reviewer #1: No	Reviewer #2: No
